# Emergence of distinct electronic states in epitaxially-fused PbSe quantum dot superlattices

**DOI:** 10.1038/s41467-022-33955-w

**Published:** 2022-11-10

**Authors:** Mahmut S. Kavrik, Jordan A. Hachtel, Wonhee Ko, Caroline Qian, Alex Abelson, Eyup B. Unlu, Harshil Kashyap, An-Ping Li, Juan C. Idrobo, Matt Law

**Affiliations:** 1 Materials Sciences Division, Lawrence Berkeley National Laboratory, Berkeley, CA USA; 2 Department of Materials Science and Engineering, University of California, San Diego, CA USA; 3 Center for Nanophase Materials Sciences, Oak Ridge National Laboratory, Oak Ridge, TN USA; 4 Department of Chemical and Biomolecular Engineering, University of California, Irvine, CA USA; 5 Department of Materials Science and Engineering, University of California, Irvine, CA USA; 6 Materials Science and Engineering Department, University of Washington, Seattle, WA USA; 7 Department of Chemistry, University of California, Irvine, CA USA

**Keywords:** Quantum dots, Electronic properties and materials

## Abstract

Quantum coupling in arrayed nanostructures can produce novel mesoscale properties such as electronic minibands to improve the performance of optoelectronic devices, including ultra-efficient solar cells and infrared photodetectors. Colloidal PbSe quantum dots (QDs) that self-assemble into epitaxially-fused superlattices (epi-SLs) are predicted to exhibit such collective phenomena. Here, we show the emergence of distinct local electronic states induced by crystalline necks that connect individual PbSe QDs and modulate the bandgap energy across the epi-SL. Multi-probe scanning tunneling spectroscopy shows bandgap modulation from 0.7 eV in the QDs to 1.1 eV at their necks. Complementary monochromated electron energy-loss spectroscopy demonstrates bandgap modulation in spectral mapping, confirming the presence of these distinct energy states from necking. The results show the modification of the electronic structure of a precision-made nanoscale superlattice, which may be leveraged in new optoelectronic applications.

## Abstract (web summary)

Self-assembled PbSe quantum dot (QD) superlattices are a class of materials that promises novel mesoscale electronic properties due to electronic coupling between individual QDs. Here, the authors reveal distinct electronic states manifested by the quantum confinement of charge carriers in epitaxially formed necking between QDs.

## Introduction

The electronic and optical properties of colloidal semiconductor quantum dots (QDs) can be controlled by tailoring their size and shape^1,2^. Their electronic structure differs from that of macroscopic crystals due to confinement of the electron and hole wavefunctions within the QD, resulting in discrete “atom-like” energy states rather than continuous bands^3,4^. Periodic modulation of quantum confinement in real space is expected to induce new features in the band structure and alter the energy landscape of a material. Modifying energy states through the quantum confinement effect has been extensively explored in several physical systems, including multilayer thin films, lithographically patterned two-dimensional electron gases in semiconductor heterostructures, and self-assembled quantum dots to realize these mesoscale phenomena and create new quantum materials^5–8^. Among these material systems, colloidal QDs have significant advantages because their size is easily tunable below 10 nm, resulting in large bandgap tunability (up to several eV, depending on the material)^9^. This energy range is highly relevant to current electronic and optical devices. However, precise control of QD size and nanoscale arrangement is critical for these applications, which imposes great experimental challenges and requires new approaches. In principle, the energy landscape of QD systems can be deliberately changed via epitaxially fusing QDs into spatially- and energetically-ordered superlattices (SL), strengthening the electronic coupling between QDs and inducing carrier delocalization throughout the solid. Inter-QD coupling in QD superlattices has been theorized to promote the emergence of electronic mini-bands that yield high carrier mobility, topological states, and Bloch oscillations^10–12^. Recent studies have focused on understanding the self-assembly and ligand exchange processes needed to synthesize highly-ordered epitaxially-fused QD superlattices (epi-SLs)^9^. Among colloidal nanocrystals, PbSe QDs are especially promising for optoelectronic applications because their large exciton Bohr radius results in strong quantum confinement and wavefunction overlap between neighboring QDs^13^. Recently, three-dimensional PbSe QD epi-SLs with micron-sized grains have been demonstrated as a system for studying mesoscale physics^14^. Optical spectroscopy studies of these materials have shown spectral changes induced by the formation of necks^14^, but such studies do not provide a microscopic picture, and it is still unknown how epitaxial fusion modifies the local electronic structure of the coupled QDs, which is crucial to understanding and controlling their mesoscopic behavior.

In this work, the local electronic structure of PbSe QD epi-SLs is probed using a combination of scanning tunneling microscopy/spectroscopy (STM/STS) and monochromated aberration-corrected scanning transmission electron microscopy/electron energy loss spectroscopy (STEM-EELS). Bandgap modulation in QD superlattices by quantum confinement has been predicted with theory^9^, however, characterizing the energy landscape at the nanometer scale is a major challenge. By utilizing nanoscale spectroscopy with STS and EELS, we correlate the local physical and electronic structure to reveal the distinct confinement of carriers in the necks compared to unfused QDs. A long-range periodic order between epitaxially-fused QDs is observed, in agreement with previous reports^15,16^. The STM-STS measurements reveal an average QD bandgap of 0.7 eV, close to the optical bandgap of the colloidal QD dispersion, while the smaller epitaxial connections (necks) have a bandgap of 1.1 eV, in good agreement with previous studies on the relationship between bandgap and PbSe QD diameter^17^. Complementary EELS measurements show that the epi-SL has an onset of energy loss around 1.1 eV and unique spectral features between 1.1 eV and ~4 eV not observed in isolated QDs (iso-QDs). The 1.1 eV bandgap energy and low energy interband transitions are consistent with electronic states induced by quantum confinement within the epitaxial necks between QDs, suggesting the emergence of distinct electronic states within the necks that change the energy landscape of the epi-SLs.

## Results and discussion

Epi-SLs formed through oriented attachment of PbSe QDs were fabricated by self-assembly and ligand exchange on the surface of liquid ethylene glycol^14^. In this work, the electronic structure of a multilayer (3D) epi-SL thin-film was investigated with STM/STS and a similarly-prepared monolayer (2D) epi-SL sample was studied with EELS. Multilayer epi-SLs were employed for STM because they have larger, more uniform SL grains and are better suited to two-probe STS experiments than QD monolayers, which have many lateral discontinuities that limit current flow. EELS measurements were performed on monolayer epi-SLs in order to simplify the imaging and image analysis. Although the fabrication and the structure of the 2D epi-SL differ from those of the 3D epi-SL, the structural repeat unit—QD/neck/QD— is similar for both samples.

The 3D epi-SL sample was fabricated by drop casting a dispersion of oleate-capped PbSe QDs (6.5 nm diameter) in hexanes onto the surface of ethylene glycol (EG), allowing the hexanes to evaporate while the QDs self-assemble on the liquid surface, and injecting a solution of 1,2-ethylenediamine (EDA) into the EG to trigger oleate removal and epitaxial fusion of the QDs. This procedure results in a polycrystalline 3D epi-SL with a distorted simple cubic unit cell. Details of the sample fabrication and structural analysis can be found elsewhere^11,14^. The 2D epi-SLs were made by drop-casting the oleate-capped PbSe QDs in toluene onto an SiO_2_ substrate and treating the monolayer with ammonium thiocyanate to remove oleate and trigger epi-fusion (see Methods).

**Fig. 1:**
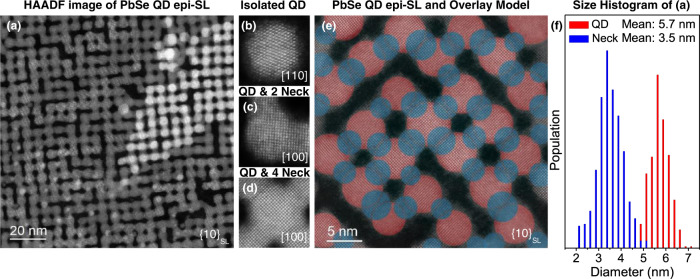
Structural analysis of the monolayer PbSe QD epi-SL. **a** HAADF-STEM image of an epi-SL showing various structural defects, including missing QDs and necks. **b**–**d** Atomic-resolution HAADF reference images of (**b**) an iso-QD, (**c**) a QD with two necks, and (**d**) a QD with four necks, demonstrating the crystallinity of the QDs and the epitaxial nature of the necks. **e** QD and neck diameters measured from a HAADF-STEM image. Red and blue circles denote QD and neck diameters, respectively. **f** Histogram of QD and neck diameters from the image. Average QD diameter: 5.7 ± 0.5 nm; average neck diameter: 3.5 ± 0.6 nm.

Atomic-resolution high-angle annular dark field (HAADF) STEM was employed to investigate the structure of the monolayer epi-SL samples. Figure 1a shows a representative large-area HAADF image of an epi-SL, which consists of a QD monolayer with a small bilayer region (high contrast sliver). Various structural defects are visible in this image, including missing QDs (vacancies), missing necks, and misoriented QDs, consistent with previous reports on these systems, which have been shown to influence the optical and electronic properties of PbSe QD superlattices^9,15^. The QDs are connected to each other in most regions of the sample (i.e., there are few iso-QDs) with varying degrees of epitaxial necking, resulting in a slightly oblique superlattice. Examples of QDs that are isolated (zero nearest neighbors (NNs)), incompletely connected (<4 NNs), and fully connected (4 NNs) are shown in Fig. 1b–d. Atomic-resolution imaging shows that the QDs are monocrystalline, in epitaxial registry with their neighbors, and fuse along the PbSe {100} facets. Representative size nonuniformity of the QDs and necks in the epi-SL is highlighted in the magnified image in Fig. 1e.

Figure 1f is a histogram of the size distribution of the QDs and necks in Fig. 1e. Details about the algorithm to determine the QD diameter are presented in Fig. S1 of the Supplementary Information. The red and blue highlighted regions in Fig. 1e denote the measured diameters of the QDs and necks, respectively, obtained from this analysis. The mean QD diameter (among 361 QDs) and neck diameter (among 531 necks) is 5.7 nm and 3.5 nm, respectively, with standard deviations of 0.5 nm and 0.6 nm, consistent with previous reports^14,15^.

The influence of inter-QD necking on the local electronic structure of the epi-SL was investigated using STM/STS. These experiments measure the differential tunneling conductance, d*I*/d*V*, which is proportional to the local density of states (LDOS); therefore, the local electronic structure can be investigated directly^18^. However, maintaining a stable tunneling condition is highly challenging for the conventional single-probe STM configuration, in which the substrate under the epi-SL is biased and the STM tip scans across the QDs. The electrical resistance between the epi-SL and substrate is moderated by residual surface ligands and band offsets, resulting in a large resistance that obstructs and destabilizes the tunneling current^19,20^. Desorption of the surface ligands by high-temperature annealing and functionalization of the conductive substrates with linkers such as dithiols is problematic on account of the heat- and air-sensitive PbSe QDs^19^. To minimize the electrical resistance between epi-SL and substrate, a sub-monolayer epi-SL can be deposited on a metallic substrate and observed by STM/STS, but the interaction between the QDs and substrate can strongly affect the electronic bands of the QDs^21,22^.

**Fig. 2:**
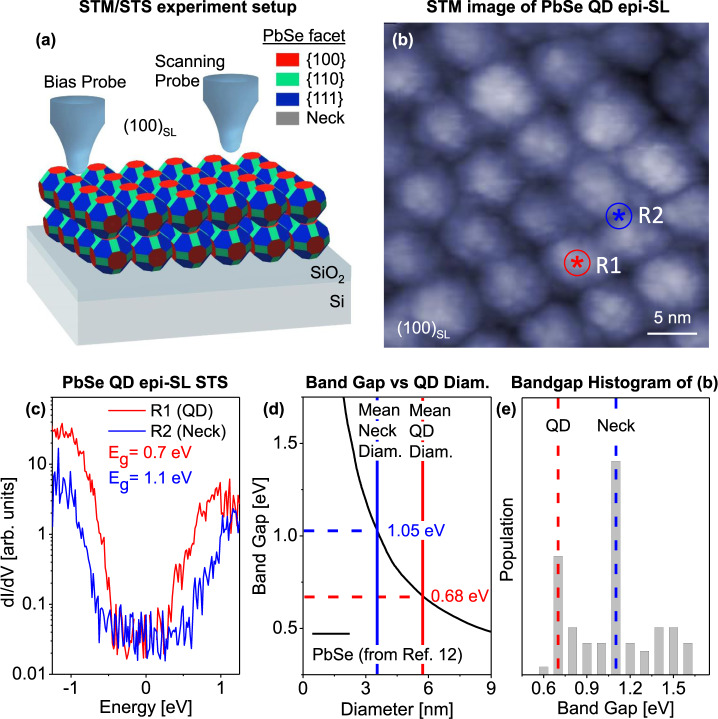
Electronic structure of multilayer epi-SLs probed by STM/STS. **a** Cartoon of two-probe STM-STS measurement of a (100)_SL_-oriented epi-SL on a SiO_2_/Si substrate (illustration is not to scale). We note that the radius of curvature of the STM tip (20-100 nm) is larger than the size of the necks and QDs (3-7 nm). Tunneling current was measured between a bias probe in soft contact with the epi-SL and a scanning probe that was kept in a tunneling position by the feedback loop. **b** A representative STM image of an (100)_SL_-oriented epi-SL grain (*V* = 3 V, *I* = 1 pA). Red and blue symbols denote nominal probe locations for STS in **c**. **c** Representative d*I*/d*V* spectra acquired from the QD center (red circle in **b**) and neck (blue circle in **b**) adjoining two QDs. Bandgaps of 0.7 eV for the QD and 1.1 eV for the neck were extracted from the spectra. **d** Dependence of QD bandgap on diameter from ref. 12. **e** Histogram of the bandgap values obtained from 81 STS locations in **b**. The histogram shows peaks at 0.7 eV and 1.1 eV, which are hypothesized to correspond to the expected bandgaps of the QDs and necks, respectively.

In this work, the above challenges were overcome by employing a two-probe STM configuration: one STM probe was placed in the tunneling position with a feedback loop to scan the sample, and the other in contact with a nearby part of the film to bias the epi-SL and collect the tunneling current from the first probe (the geometry is shown schematically in Fig. 2a with additional detail in Fig. S2)^23,24^. This configuration enables effective STS measurements of the epi-SL in the shell-tunneling regime (no carrier accumulation), since the tunneling current across the epi-SL bypasses the epi-SL-substrate barrier^25^. Therefore, the spectral features correspond to the energy levels of the epi-SL. We note that the ST spectra were measured in the shell-tunneling regime by keeping the tunneling current low to ensure that charging is minimal (10 meV)^25^. The impact of ligands on the electronic structure of the epi-SL and the electrical current should be negligible in these experiments because the oleate and glycoxide ligands are electrically insulating and located only in the free volume of the film away from the necks. We therefore believe that the current flows entirely through the inorganic network of necked QDs.

Figure 2b shows an STM image acquired on a (100)_SL_-oriented 3D epi-SL (6-7 QD layers, ~40 nm thick), demonstrating the ability to resolve individual QDs and necks directly from the tunneling current. This STM image was used as a reference to measure the LDOS at the centers of the QDs and the midpoints of the necks. The influence of necking on the LDOS is observed by comparing d*I*/d*V* spectra of a QD (R1-red circle) and a neck (R2-blue circle), which are shown in Fig. 2c. Each spectrum is an average of ten spectra taken at the same location. In the QD spectrum (R1), the tunneling current diminishes to noise level between −0.35 eV and 0.35 eV, indicating an electronic bandgap of 0.7 eV, while the STS from a neck (R2) shows a 1.1 eV bandgap, distinctively larger than that of the QD. The electronic bandgap of a PbSe QD is inversely proportional to its diameter due to its nearly linear dispersion around the bandgap, which has been determined by Moreels et al., as shown in Fig. 2d^17^. By overlaying the average QD and neck diameter values that were established in the histogram shown in Fig. 1f, a bandgap value of 0.68 eV is observed for the QDs and 1.05 eV is observed for the necks according to Fig. 2d. These values match well with the bandgaps of both the QDs and necks in the STS measurements from Fig. 2c. The larger bandgap of the necks relative to QDs was consistently observed across the epi-SL surfaces in various conditions, including different surface orientation of the epi-SL and surface treatments (Figs. S2, S3 in the Supplementary Information).

While the inverse correlation between QD bandgap and average diameter is expected, observing a similar relation for the necks is notable. This suggests the confinement of carriers and emergence of new electronic states in the necks similar to those present in the QDs. More critically, the tunneling spectra of both the QDs and the necks show not discrete energy levels like those of a single iso-QD^13^ or monolayers of epitaxially-fused QDs^17^, but rather a smoothly increasing LDOS above the band edges. This difference may arise from several factors. First, the experiment temperature used here (80 K) is substantially higher than in previous studies (5 K), which can thermally broaden the LDOS^26–28^. Seconds, the samples were prepared and measured in a different way. In contrast to previous reports, our samples were not subjected to chemical or thermal treatments to remove ligands, hence we measure pristine ligand-capped samples in this work. In addition, the degree of quantum coupling is different between the epi-SL samples studied in this work and in previous reports. Here, the STS was performed on 3D epi-SLs in which the QDs are connected to six neighbors with ~80% connectivity, causing additional coupling that can smear out the resonance peaks.

To gain statistical evidence of the distinct bandgaps between QDs and necks, we performed STS measurements at equally spaced grid positions over the 35 × 35 nm area shown in Fig. 2b. The bandgap at each of the 81 nominal probe positions is tabulated in the histogram in Fig. 2e. The histogram shows a distribution of bandgaps between 0.6 eV and 1.6 eV with sharp peaks at 0.7 eV and 1.1 eV, in agreement with the values of the QD and neck bandgaps in Fig. 2d, respectively. It should be noted that it is not possible to rigorously associate the bandgaps from the STS grid with the probe position due to the spatial drift of the tip and the tip convolution caused by its comparably large radius of curvature (the typical tip radius of curvature is 20-100 nm, significantly larger than the feature size of 3-7 nm)^29,30^. Furthermore, there is no distinct feature in Fig. 2c from the hollows because when the probe goes down into the hollow, the tunneling occurs between the side of the STS probe and the side of the QDs. From this histogram, the mean value of the bandgap for QDs and necks is 0.72 ± 0.05 eV and 1.2 ± 0.2 eV. The histogram maxima at the QD and neck bandgaps are consistent with some significant fraction of the film having a bandgap of 1.1 eV, which we attribute to the necks (the number of necks is about twice the number of QDs, as seen in Fig. 1). This corroborates the hypothesis that the necks act as more confined regions of the QD film and introduce new states into the electronic structure of the epi-SL.

**Fig. 3:**
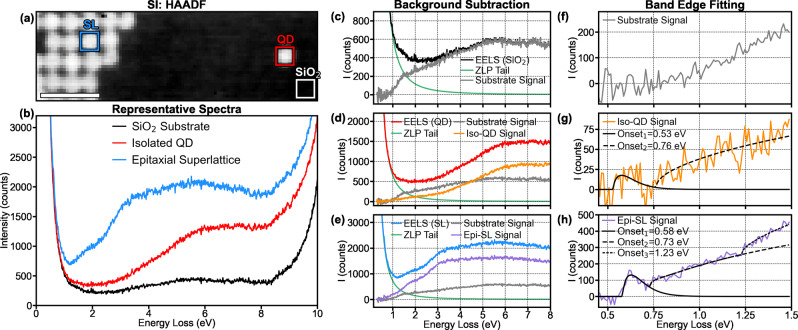
EELS identification of distinct electronic states in the epi-SLs. **a** HAADF reference image from the SI acquired of a region with a single iso-QD in proximity to an area of epi-SL. **b** Three representative spectra from different regions in the spectral image representing the SiO_2_ substrate signal, an iso-QD, and the epi-SL. The pixels used for the representative spectra are labeled in **a**. **c**–**e** The representative spectra from (**b**) with a power law background subtraction to remove the elastic scattering (ZLP) tail, revealing the shape of the signal from the SiO_2_ substrate (**c**), the iso-QD (**d**), and the epi-SL (**e**). For the iso-QD and epi-SL spectra, we also subtracted the substrate signal, as the beam interacts with the substrate at all locations. The substrate signal is shown in (**f**) as a reference. We then fit band-edge functions to the iso-QD (**g**) and epi-SL signals (**h**). The fits show an onset of intensity at ~0.55 eV in both the iso-QD and the epi-SL (but not in the substrate signal) and a second onset at ~1.23 eV only in the epi-SL signal.

The electronic structure of QDs can be investigated using EELS, which has been previously reported only on iso-QDs^31,32^. Here, the local electronic structure of a monolayer PbSe QD epi-SL was investigated with EELS in a monochromated aberration-corrected STEM^33^. In Fig. 3, a hyperspectral EELS analysis was performed on a region possessing an iso-QD next to a section of monolayer epi-SL (with necking and >0 NNs). An EEL hyperspectral dataset is called a spectrum image (SI), meaning an EEL spectrum is acquired at each probe position in a 30 × 95 grid of pixels to create a three-dimensional dataset with two spatial dimensions and one spectral dimension. The HAADF image in Fig. 3a is simultaneously acquired with the SI, allowing us to highlight specific pixels based on the structure and directly plot their representative spectra. The pixels selected from the spectra for the bare substrate (labeled SiO_2_), the iso-QD, and the epi-SL are shown in Fig. 3b.

In all three spectra, the higher energies are dominated by the band edge of the supporting SiO_2_ substrate (>8.5 eV), which is expected since the substrate is uniformly present everywhere in the region. Also common to all three spectra is the tail of the elastic scattering or zero-loss peak (ZLP) at ultralow energies. The tail is a function of the instrumental condition, not the sample, and is therefore nearly identical in all three spectra. Figure 3c–e show a power law background subtraction to remove the effect of the ZLP tail to reveal the real signals associated with the SiO_2_, iso-QD, and epi-SL spectra, respectively.

Additionally, we must contend with the presence of carbon contamination that accumulates on the sample during fabrication and handling and cannot be completely removed due to the sensitivity of the sample to standard cleaning techniques (such as plasma cleaning). As a result, a significant non-zero EELS intensity is observed in the substrate signal even far below the 8.5 eV bandgap of SiO_2_. Thus, the substrate signal is also subtracted from the spectra to reveal the true iso-QD (Fig. 3d, g) and epi-SL (Fig. 3e, h) signals.

With the electronic structure isolated from instrumental and environmental artifacts, we can apply band-edge fitting to the onset of the EELS intensity in the PbSe QDs. Here, we use the fitting model for direct bandgaps suggested by Zamani et al. to fit onsets in the EEL intensity^34^. EELS probes the electronic structure by measuring the energy loss associated with transitions from the valence band to the conduction band, making it analogous to the joint density of states (JDOS)^33^. Therefore, interband excitations do not manifest as peaks, but rather as onsets of EEL intensity at the bandgap energy. For instance, EEL intensity associated with the SiO_2_ bandgap shows a maximum at 10.5 eV although the SiO_2_ bandgap is 8.5 eV. It is also important to note, that for this complex multi-gap band structure, the EEL intensity captures not only the onsets due to bandgaps, but also changes in the interband transition density. As a result, we used multiple bandgap functions to fit the band-edge electronic structure even if only a single bandgap was present. For the iso-QD signal, a two-function fit was observed to produce the best fit (with onsets at 0.53 eV and 0.76 eV). For the epi-SL signal, a three-function fit was with required, with onsets at 0.58 eV, 0.73 eV and 1.23 eV.

The band-edge fitting corroborates the analysis in STS of two distinct bandgaps existing in the QDs and necks. Both bandgaps should be observed in the epi-SL, as both QDs and necks are present, but only the QD bandgap should be observed in an iso-QD. Here, the ~0.55 eV and ~0.75 eV onsets are observed in both the iso-QD and epi-SL signals, while the 1.23 eV onset is only observed in the epi-SL. Thus, the lower energy onsets correspond to the QDs with a measured bandgap of ~0.55 eV (the ~0.75 eV onset is likely a change in the interband transition density), and the high-energy onset corresponds to the neck electronic structure with a measured bandgap of 1.23 eV. Further validation and discussion of the band-edge fitting is included in Figs. S4 and S5.

We note that there is some deviation between the bandgap values derived from the EELS modeling and STS measurements. The STS and EELS are performed on different samples, so some deviation is reasonable, and more critically there are some limitations in the accuracy of the EELS measurement of the exact bandgap values (which are addressed in the supplementary discussion). Second, the bandgap determined from STS is equal to the sum of single particle gap and the self-energy of electrons and holes^35^, while the bandgap from EELS is more closely related to the bandgap from the JDOS^36^. However, these differences should be negligible due to the high dielectric constant of PbSe.

There is a strong consistency in the electronic structure from 0.5-1.2 eV between the epi-SL and iso-QD signal, which indicates that they have similar electronic structure. Moreover, the band-edge fitting clearly shows a new onset in the epi-SL that is not observed in the iso-QD, at an energy that is quite close to the STS measurement of the QD neck bandgap. Although it is difficult to directly measure bandgaps in the QD necks with EELS, the presence of a distinct bandgap can be unambiguously inferred from the comparison of the epi-SL and iso-QD signals.

These new electronic states are a universal aspect of the epitaxially-connected QD system. By examining other samples, we observe that the additional onset of EEL intensity in Fig. 3 is highly repeatable and, more importantly, a fundamental aspect of the electronic structure of epi-SLs (as opposed to a surface effect such as a plasmon or a polariton). To visualize these new electronic states and compare them across multiple datasets and sample regions, a component separation technique called non-negative matrix factorization (NMF) was utilized. NMF decomposes the SIs into a set number of components, each with a spectral endmember and a spatial abundance map, where the linear combination of each endmember at the intensity of the abundance maps reconstructs the original dataset. For simple systems, the spectral endmembers correspond directly to spectral phenomena in the system with different localizations, and the spatial abundance maps provide a visualization of the location and intensity where these phenomena occur.

**Fig. 4:**
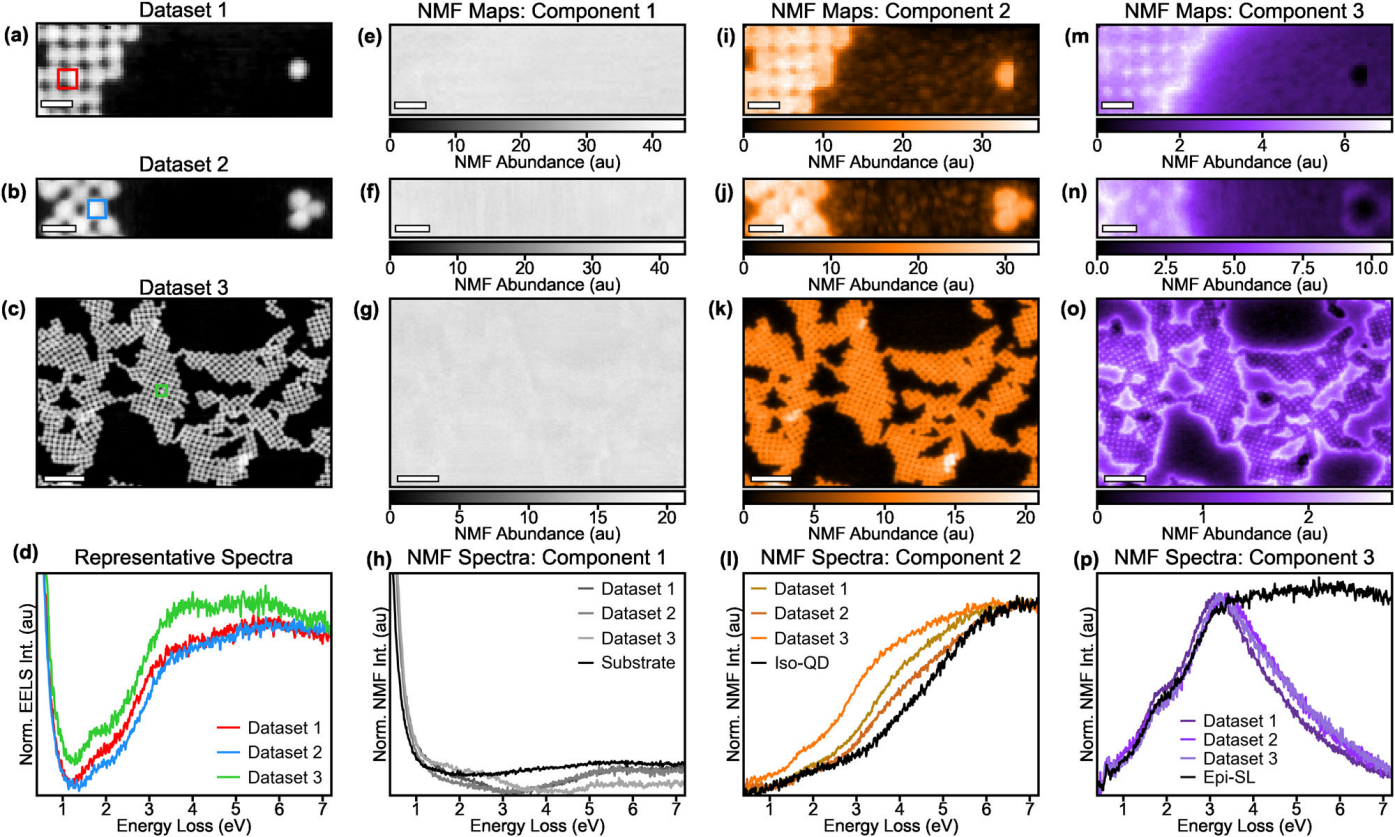
Realization of bandgap modulation in epi-SLs with NMF analysis. Three EEL SIs of QDs with no necks compared to the epi-SLs with necking. **a**–**c** HAADF STEM reference images of the three datasets. **a** Shows the dataset with only a single iso-QD located near the epi-SL from the Fig. 3. **b** Shows a trimer cluster without necking, (**c**) shows a large area epi-SL containing various necking configurations. **d** The representative spectra from the marked regions in **a**, **b**, and **c** showing that the electronic structures of the three epi-SLs is nearly identical. NMF decomposition and visual representation of the SI shown in **e**–**p**. **e**–**g**, **i**–**k** and **m**–**o** present the abundance maps showing the localization of the substrate signal, iso-QD, and epi-SL signals from regions in **a**, **b** and **c**, respectively. **h**, **l**, **p** show the corresponding NMF spectral endmembers. The maps show that new electronic states are generated by the epitaxial connections in the epi-SL. The new neck electronic states (component 3) at low energies are repeatably observed, indicating that the band-edge structure is a fundamental feature of the epitaxial necks.

To demonstrate the repeatability and spatial localization of the new electronic states emerging from the necks, SI datasets from various regions of the PbSe epi-SL were studied with a three-component NMF decomposition in Fig. 4. The simultaneously acquired HAADF images of the SI datasets are presented in Fig. 4a–c. We take representative spectra from the epi-SL in each dataset and plot them together in Fig. 4d. The band-edge structure of all three spectra is nearly identical, with peaks and changes in intensity at the same spectral values, and each shows an uptick in EELS signal at ~1.1-1.2 eV. The maps and spectra of the NMF decomposition analysis for all three datasets are shown in Fig. 4e–p, and we observe highly similar behavior between the three datasets despite widely varying geometries and topographies. The first component captures the tail of the ZLP and the SiO_2_ substrate. The second component captures the iso-QD electronic structure. The third component captures the new electronic states in the epi-SL due to the QD necks.

The abundance maps of the first NMF component (ZLP and substrate) are presented in Fig. 4e–g, which show uniform contrast corresponding to the uniform beam condition and membrane/ligands/contamination. The spectral endmembers are plotted in Fig. 4h, which match well with the representative SiO_2_ (substrate) spectra from Fig. 3. The abundance maps of the second component are shown in Fig. 4i–k, and they demonstrate that these electronic states are observed everywhere regardless of the presence of epitaxial necking. Moreover, the second component spectral endmembers (Fig. 4l) match the iso-QD signal from Fig. 3, confirming that this component represents the electronic structure of unfused PbSe QDs. Lastly, the maps of the third component (new electronic states of the epi-SL) are shown in Fig. 4m–o. In datasets 1 and 2, these new electronic states are visible in the epi-SL but not in either the iso-QD in Fig. 4m or the un-necked trimer of iso-QDs in Fig. 4n, illustrating the presence of this component requires QD necking.

We also note that the component 3 spectral endmembers for all three datasets in Fig. 4p show nearly the same band-edge electronic structure as the epi-SL signal from Fig. 3. The similarity between NMF components illustrates that the EEL intensity in the spectral regime is a fundamental feature of the electronic structure of the epi-SLs. Since collective surface phenomena (such as plasmons and polaritons) are highly geometry dependent, the large structural differences between the epi-SLs in the three datasets would result in significant variations frequency and line-shape in the spectrum for surface effects, but not for electronic structure effects. Perhaps more importantly, we note that at higher energies the NMF component 3 spectrum differs from the epi-SL signal. However, these are the energies where NMF component 2 has a high intensity, indicating that, while component 2 represents the iso-QD electronic structure directly, component 3 represents the new electronic structure induced by the epitaxial necking only. Thus, the EELS epi-SL signal is shown to be a linear combination of iso-QD signal (component 2) and the epitaxial neck signal (component 3). A closer examination of the band-edge electronic structure in the raw-spectral analysis of Fig. 3 and the NMF decomposition in Fig. 4 is shown in Fig. S6.

One of the most notable aspects of the epi-SL component, in the large-area dataset 3, is the presence of dead spots where the epi-SL states are significantly suppressed compared to the surrounding regions. Furthermore, while some of these dead spots occur in areas that exhibit irregular structure in the corresponding HAADF image of Fig. 4c, some appear in places that are nominally uniform. This implies that poor necking or other nanoscale/atomistic heterogeneity can influence the formation of these new electronic states.

As further validation, we conducted off-axis EELS measurements from the epi-SLs and isolated QDs. By collecting EELS many tens of milliradians away from the optic axis of the electron microscope, the contribution from the delocalized dipole scattering is minimized, facilitating the identification of highly-localized emergent properties^37^. The measurements shown above were performed on-axis to maximize the signal to noise, but Fig. S8 shows that the highly-localized off-axis EELS analysis identifies the same spectral signatures as modeled above in Fig. 3. Furthermore, we also performed the same band-edge fitting from Fig. 3 on dataset 2 from Fig. 4 (the trimer cluster of QDs without epitaxial necking) and achieve an excellent match (Fig. S9). These additional experiments further corroborate the conclusion that the epitaxial necks induce a new electronic structure in the PbSe QD films.

This work reports direct measurements of the local electronic structure of PbSe QD epi-SLs. Structural analysis with STEM-HAADF imaging affirmed the presence of epitaxial necks between QDs in the epi-SL structure. The local density of states measured with STS revealed the modulation of the bandgap energy from 0.72 ± 0.05 eV in the QDs to 1.1 ± 0.2 eV in the necks. Distinct electronic states induced by the necks were confirmed with complimentary EELS. Decomposition of the EELS data with NMF analysis revealed electronic signatures of necking within the epi-SL. The larger bandgap of the relatively smaller necks (effective diameter of 3.5 nm) is experimental validation of the anticipated spatial periodicity of quantum confinement in QD epi-SLs. The relatively small potential barriers for electrons and holes may result in strong electronic coupling and the emergence of electronic mini-bands in sufficiently ordered samples, as predicted by theory^38,39^. This work shows that the application of high spatial and energy resolution techniques such as STS and EELS can directly visualize the energy landscape of QD superlattices sensitive to local disorder. The distinct energy levels of the necks reshape the energy landscape for charge carriers and will be an important consideration in the design of future optoelectronic devices from QD epi-SLs.

## Methods

### Materials

All chemicals were used as received unless otherwise noted. Lead oxide (PbO, 99.999%), lead iodide (PbI_2_, 99.9985%) and selenium shot (99.999%) were purchased from Alfa Aesar. Oleic acid (OA, technical grade, 90%), diphenylphosphine (DPP, 98%), 1-octadecene (ODE, 90%), anhydrous ethylene glycol (EG, 99.8%), anhydrous acetonitrile (99.99%), anhydrous hexanes (99%), anhydrous toluene (99.8%), 3-mercaptopropyltrimethoxysilane (3-MPTMS, 95%), trimethylaluminum (TMA, 97%), ammonium thiocyanate (NH_4_SCN, 99.99%) and anhydrous dimethyl sulfoxide (DMSO, 99.9%) were purchased from Sigma Aldrich. Anhydrous 1,2-ethylenediamine (EDA, > 98.0%) was purchased from TCI. Trioctylphosphine (TOP, technical grade, >90%) was acquired from Fluka and mixed with selenium shot for 24 h to form a 1 M TOP-Se stock solution. 18.2 MΩ water (Milli-Q Gradient) was used for substrate cleaning and atomic layer deposition (ALD). Water for ALD was degassed with three freeze-pump-thaw cycles before use.

### Quantum dot synthesis

PbSe QDs were synthesized and purified using standard air-free techniques according to a previously published procedure^14^. Briefly, PbO (1.50 g), OA (5.00 g), and ODE (10.00 g) were mixed and degassed in a three-neck round-bottom flask at room temperature. The mixture was then heated at 120 °C under vacuum to form Pb(OA)_2_ and dry the solution. After 1.5 h, the Pb(OA)_2_ solution was heated to 180 °C under argon flow and 9.5 mL of a 1 M solution of TOP-Se containing 200 µL of DPP was rapidly injected into this hot solution. An immediate darkening of the solution was observed, and the QDs were grown for 105 s at ~160 °C. The reaction was quenched with a liquid nitrogen bath and injection of 10 mL of anhydrous hexanes. The QDs were purified in an N_2_-filled glovebox (<0.5 ppm O_2_) by adding 2 mL of toluene and 9 mL of acetonitrile to the reaction solution, collecting the QDs by centrifugation, performing 6–8 cycles of redispersion/precipitation using toluene/acetonitrile (3 mL/24 mL), and then drying and storing the QDs as a powder in the glovebox.

### Fabrication of multilayer QD superlattices for STM/STS experiments

Multilayer (3D) epi-SL films consisting of 6–7 QD layers (~40 nm thick) were made using a modified version of a published procedure^14^. Briefly, in an N_2_-filled glovebox with <0.5 ppm O_2_, oleate-capped PbSe QD superlattices were prepared by carefully drop casting 60 μL of a 15 mg/mL solution of PbSe QDs dispersed in hexanes onto 7 mL of ethylene glycol in a Teflon well (3.5 cm wide × 5 cm long × 1 cm deep) that was cleaned by soaking overnight in 5 M nitric acid and then soaking and rinsing in Millipore water at least ten times. After depositing the QD solution, the well was immediately covered with a glass slide and the hexanes allowed to slowly evaporate in 25–30 min, resulting in a smooth, dry QD film floating on the EG surface. The slide was then removed and 100 μL of a 7.5 M solution of ethylenediamine in acetonitrile was slowly (5–10 s) injected into the EG directly underneath a corner of the film. As the EDA solution spread throughout the well, the film visibly darkened, indicating film densification and conversion to the epi-SL. After 30 s, the darkened epi-SL film nearest to the EDA injection point was transferred onto a SiO_2_/Si (30 nm thermal SiO_2_) substrate by manual stamping using a vacuum wand. Prior to stamping, the SiO_2_-coated Si substrate was cleaned by 15 min of sonication in acetone, water, and isopropanol, dried under flowing air, soaked in a 100 mM solution of 3-MPTMS in toluene for 1 h to functionalize the surface for improved QD adhesion, then rinsed with neat toluene and dried under flowing air. The stamped film was rinsed vigorously with neat acetonitrile and dried under flowing N_2_. Next, the epi-SL film was immediately soaked in a 10 mM solution of PbI_2_ in DMSO for 5 min to reduce the amount of residual oleate and adsorbed glycoxide, rinsed with copious amounts of DMSO and acetonitrile, and dried under flowing N_2_.

### Fabrication of monolayer QD superlattices for STEM EELS experiments

Monolayer (2D) epi-SL films for EELS experiments were made as follows. An aliquot of a 1.75 mg/mL PbSe QD dispersion in toluene was drop cast onto a Pelco SiO_2_ support film TEM grid (8 nm thick SiO_2_ windows, product 21532-10, Ted Pella) and dried over the course of several minutes. The grid was then immersed for 15 s in a 15 mM ammonium thiocyanate (NH_4_SCN) solution in acetonitrile, soaked in neat acetonitrile for 10 s to remove unbound NH_4_SCN, dried, soaked in neat hexanes for 10 s to remove residual unbound oleic acid/oleate, dried again, and finally dipped into neat acetonitrile, quickly removed, and blown dry in a stream of N_2_ to prevent solvent droplets from depositing residue onto the grid surface.

### STM-STS analysis

A cryogenic four-probe scanning tunneling microscope (4P-STM) from RHK/UNISOKU at Oak Ridge National Laboratory was used to measure in situ two-probe STM/STS in multilayer epi-SLs. Measurements were performed in UHV (5 × 10^−10^ Torr) at both 82 K and 300 K. Epi-SL samples were transferred from a glovebox (<0.1 ppm O_2_ and <1 ppm H_2_O) to the UHV chamber using a nitrogen-filled bag and a small glovebox that enclosed the load-lock. In situ SEM imaging in the STM was used to identify epi-SL grains and precisely locate the STM probe tips on the sample. For STM/STS measurements, one of the probes was pressed a few nm into the sample to make a good electrical contact to the epi-SL and then the bias voltage was applied, while the other probe was used to scan the sample with tunneling current kept constant with the feedback loop. The QDs in the multilayer films that were used for STM/STS measurements are necked in all three dimensions. The STM current flowed from the STM tip to the film (out-of-plane), then horizontally through the film (in-plane), then from the film to the other STM tip. However, regardless of the current path, STM probed the LDOS underneath the scanning tip because the tunneling resistance is larger than other resistances along the current path. Conventional lock-in techniques were used to measure the d*I*/d*V* spectra, where the modulation frequency was 1 kHz and the modulation voltage 30 mV. The energy conversion from the tip-bias voltage applied in STS measurements was obtained with correction factor of 0.8 (from tip-induced band banding) derived previously^19^. With this consideration, the QD below the tip is under nearly the same potential as the scanning probe since PbSe QD has a large dielectric constant (Diameter = 4 nm, *ε* = 227). Therefore, the lever arm fraction (the ratio of the voltage drop between STM tip - QD over QD - second STM probe), *η* = *V*_t_/*V* is close to unity^19^. 

### STEM-EELS analysis

STEM-EELS experiments were performed on the monolayer epi-SL samples in a double aberration-corrected, monochromated Nion Ultra-HERMES STEM microscope^40^ equipped with a Nion Iris spectrometer operating at 60 keV and an energy resolution of 100 meV at Oak Ridge National Laboratory. This energy resolution was selected as the best balance of resolution and signal (beam current). EEL spectra were acquired using a ~60 pA beam current and a convergence angle of 30 mrad through a 1 mm aperture, corresponding to a 25 mrad collection angle using Hamatsu ORCA SCMOS detector Multiple EEL spectra were acquired and summed to a single spectrum using sub-pixel alignment to maximize the signal-to-noise ratio. A post-processing normalization routine was used to account for the significant difference in the inelastic signal when the probe was passing through the QDs *vs*. only the substrate (Fig. S7). Cherenkov radiation resulting from electrons having velocities greater than the speed of light in the material, is a serious concern for this type of low-loss analysis but it is avoided by using a 60 keV electron beam, as reported previously^31,32^.

Important aspect of the EELS acquisition, significant intensity is observed outside of the epi-SL itself (especially for the neck states). This phenomenon is called the ‘aloof’ effect which allows the evanescent field of the electrons to interact with the sample non-locally, causing significant intensity outside of the PbSe. This likely accounts for much of the difference in signal intensity between the epi-SL and isolated QD signals. We verified that the effect is only quantitative by performing a technique called Off-Axis EELS, where the delocalized ‘aloof’ interaction is excluded from the collected signal. We used this technique to show that ‘aloof’ excitation does not qualitatively change the signals observed with on-axis EELS in (Fig. S8).

## Supplementary information

Supplementary Information

## Data Availability

The datasets collected in this work are available from the corresponding author on reasonable request.

## Code availability

The code used for the developed algorithm in this work are available from the corresponding author on reasonable request.
