# Author Correction: Emergence of distinct electronic states in epitaxially-fused PbSe quantum dot superlattices

**DOI:** 10.1038/s41467-022-35150-3

**Published:** 2022-11-30

**Authors:** Mahmut S. Kavrik, Jordan A. Hachtel, Wonhee Ko, Caroline Qian, Alex Abelson, Eyup B. Unlu, Harshil Kashyap, An-Ping Li, Juan C. Idrobo, Matt Law

**Affiliations:** 1 Materials Sciences Division, Lawrence Berkeley National Laboratory, Berkeley, CA USA; 2 Department of Materials Science and Engineering, University of California, San Diego, CA USA; 3 Center for Nanophase Materials Sciences, Oak Ridge National Laboratory, Oak Ridge, TN USA; 4 Department of Chemical and Biomolecular Engineering, University of California, Irvine, CA USA; 5 Department of Materials Science and Engineering, University of California, Irvine, CA USA; 6 Materials Science and Engineering Department, University of Washington, Seattle, WA USA; 7 Department of Chemistry, University of California, Irvine, CA USA

**Keywords:** Quantum dots, Electronic properties and materials

Correction to: *Nature Communications* 10.1038/s41467-022-33955-w, published online 10 November 2022

In this article the affiliation details for Jordan A. Hachtel, Wonhee Ko and An-Ping Li were incorrectly given as “Materials Science and Engineering Department, University of Washington, Seattle, WA, USA” but should have been “Center for Nanophase Materials Sciences, Oak Ridge National Laboratory, Oak Ridge, TN”.

The original article and Supplementary file have been corrected.

