# Supplementary material for: Emergence of distinct electronic states in epitaxially-fused PbSe quantum dot superlattices
Source: Nat Commun. 2022 Nov 10;13:6802. doi: 10.1038/s41467-022-33955-w (PMC9649673; doi:10.1038/s41467-022-33955-w)
Supplement: Supplementary file 1 — Supplementary Information [file 41467_2022_33955_MOESM1_ESM.pdf]

Emergence of Distinct Electronic States in Epitaxially-Fused PbSe Quantum Dot Superlattices

Mahmut S. Kavrik[1,2*]†, Jordan A. Hachtel[3*]†, Wonhee Ko[3*]†, Caroline Qian[4], Alex Abelson[5], Eyup B. Unlu[2], Harshil Kashyap[2], An-Ping Li[3], Juan C. Idrobo[6], Matt Law[4,5,7]†

[1]Materials Sciences Division, Lawrence Berkeley National Laboratory, Berkeley, CA, US

[2]Department of Materials Science and Engineering, University of California, San Diego, CA, US

[3]Center for Nanophase Materials Sciences, Oak Ridge National Laboratory, Oak Ridge, TN, US

[4]Department of Chemical and Biomolecular Engineering, University of California, Irvine, CA,US

[5]Department of Materials Science and Engineering, University of California, Irvine, CA, US

[6]Materials Science and Engineering Department, University of Washington, Seattle, WA, US

[7]Department of Chemistry, University of California, Irvine, CA, US

†Corresponding authors: Mahmut S. Kavrik, (mkavrik@lbl.gov); Jordan A. Hachtel (hachtelja@ornl.gov); Wonhee Ko, (kow1@ornl.gov); Matt Law, (matt.law@uci.edu)

[*] Indicated authors contributed equally.

Notice: This manuscript has been authored by UT-Battelle, LLC, under Contract No. DE-AC0500OR22725 with the U.S. Department of Energy. The United States Government retains and the publisher, by accepting the article for publication, acknowledges that the United States Government retains a non-exclusive, paid-up, irrevocable, world-wide license to publish or reproduce the published form of this manuscript, or allow others to do so, for the United States Government purposes. The Department of Energy will provide public access to these results of

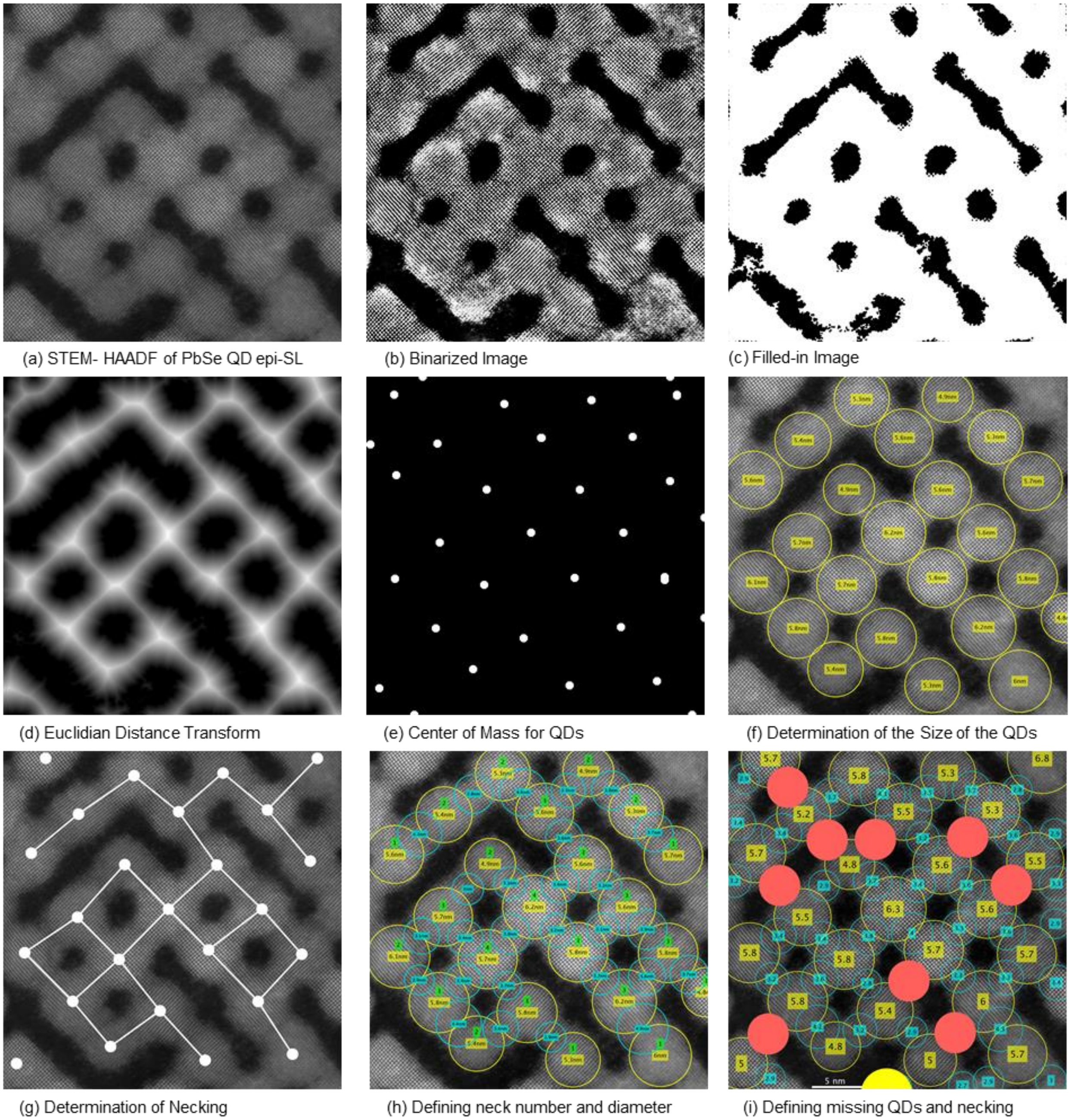

federally sponsored research in accordance with the DOE Public Access Plan (http://energy.gov/downloads/doe-public-access-plan).

Fig. S1 Structural analysis of 2D PbSe QD epi-SL from STEM-HAADF image with algorithm build in house. Position, diameter, and necking number of the QDs and necks analyzed and presented in nine steps. (a) High resolution STEM-HAADF image of the PbSe QD epi-SL display atomic resolution (b) Binarization of the image, converting grayscale image to the binary image using a combination of the global and adaptive local thresholding algorithms (c) Filling the space between atoms, morphologically dilating the image to make sure QDs are fully captured in the binary image. (d) Euclidean distance transformation employed to define the center of QDs. (e)

Positions of the QDs found by determining local maxima of the distance transform matrix which shows center of mass for QDs (f) Diameters of the QDs determined by the magnitudes of the local maxima. Circumference of the QD shown with yellow circles and diameter is denoted with text highlighted in yellow. (g) Necking between the QDs determined by investigating zero-pixel value on the line drown between center of neighboring QDs. (h) Neck positions determined by mid-point of neighboring QDs, and the diameter of the necks defined by distance transform at the center of necks. Circumference of the necks shown with blue circles and diameter is denoted with text highlighted in blue. Number of necking per QD denoted by numbers highlighted in green. (i) Missing QDs shown with yellow disk determined by Euclidean distance transform similar process used in c for inversed image. Missing necks are denoted by red disk defined by difference between possible necking and total number of necking for each QDs. For observance of a missing neck, the angle between neighboring existing necking for host QDs checked and 10 degree of tolerance is defined with one neck.

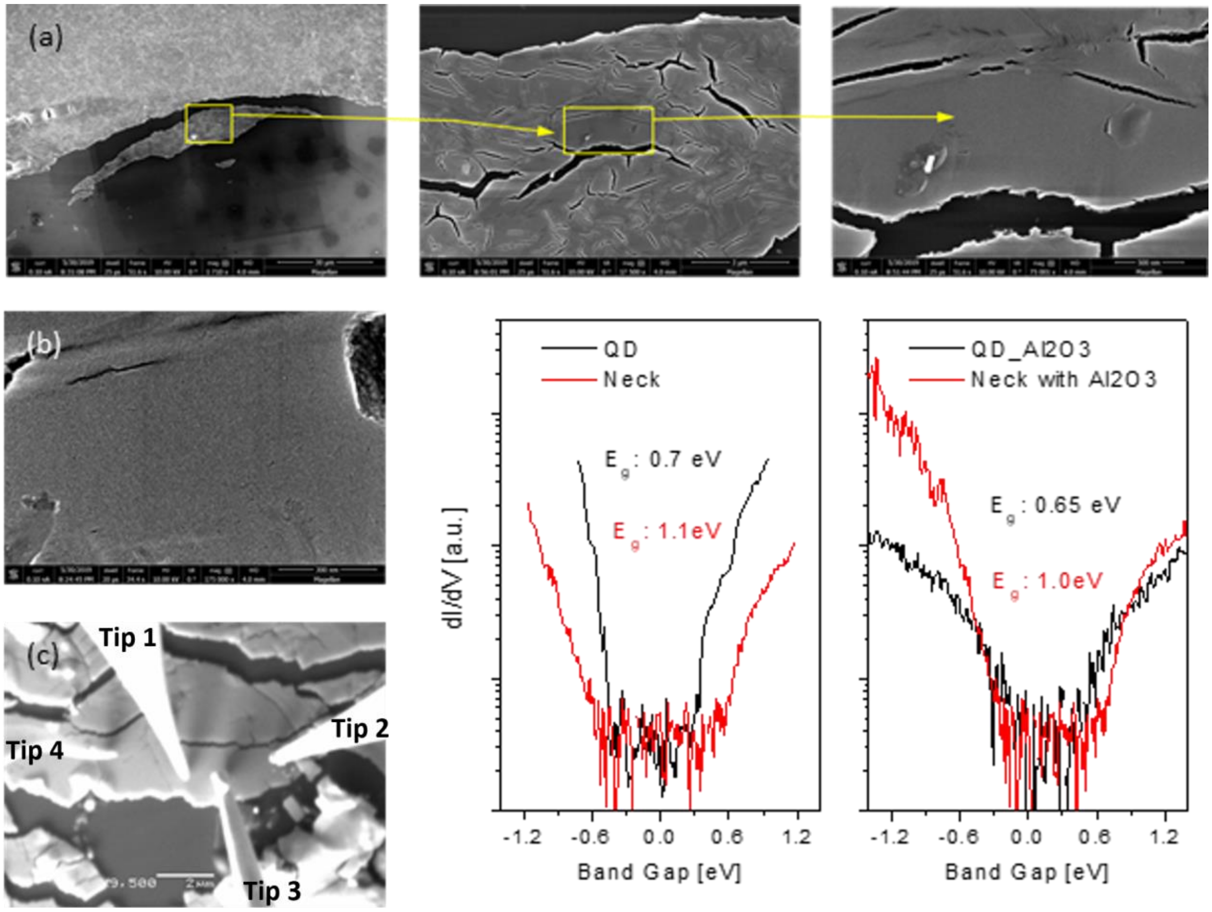

Fig. S2 STM experiment setting. (a) SEM image of the region studied with STM-STS. Bias and scanning probes. (b) High magnification SEM image of the STM location. The traces of the embedded STM probe forms seen in SEM image. (c) SEM image shows four probes available for STM/STS experiments. (d) dI/dV spectra for PbSe QD epi-SL on bare surface and with Al_2O_3 (1nm) coating. Fermi level is shifted towards the valence band with Al_2O_3 coating. Similar bandgaps were observed for QD and its necking in both samples. ***Atomic layer deposition.*** Amorphous alumina deposition was performed in a homemade cold wall traveling wave ALD system within a glovebox using trimethylaluminum and water at a substrate temperature of 54 °C and a base pressure of 0.133 Torr. Precursors were introduced to the ALD chamber using computer-controlled diaphragm valves in-line with a 250 sccm stream of nitrogen carrier gas. Pulse times were 25 ms and 30 ms for the metal and water precursors, respectively, and the purge time was 65 s for both precursors.

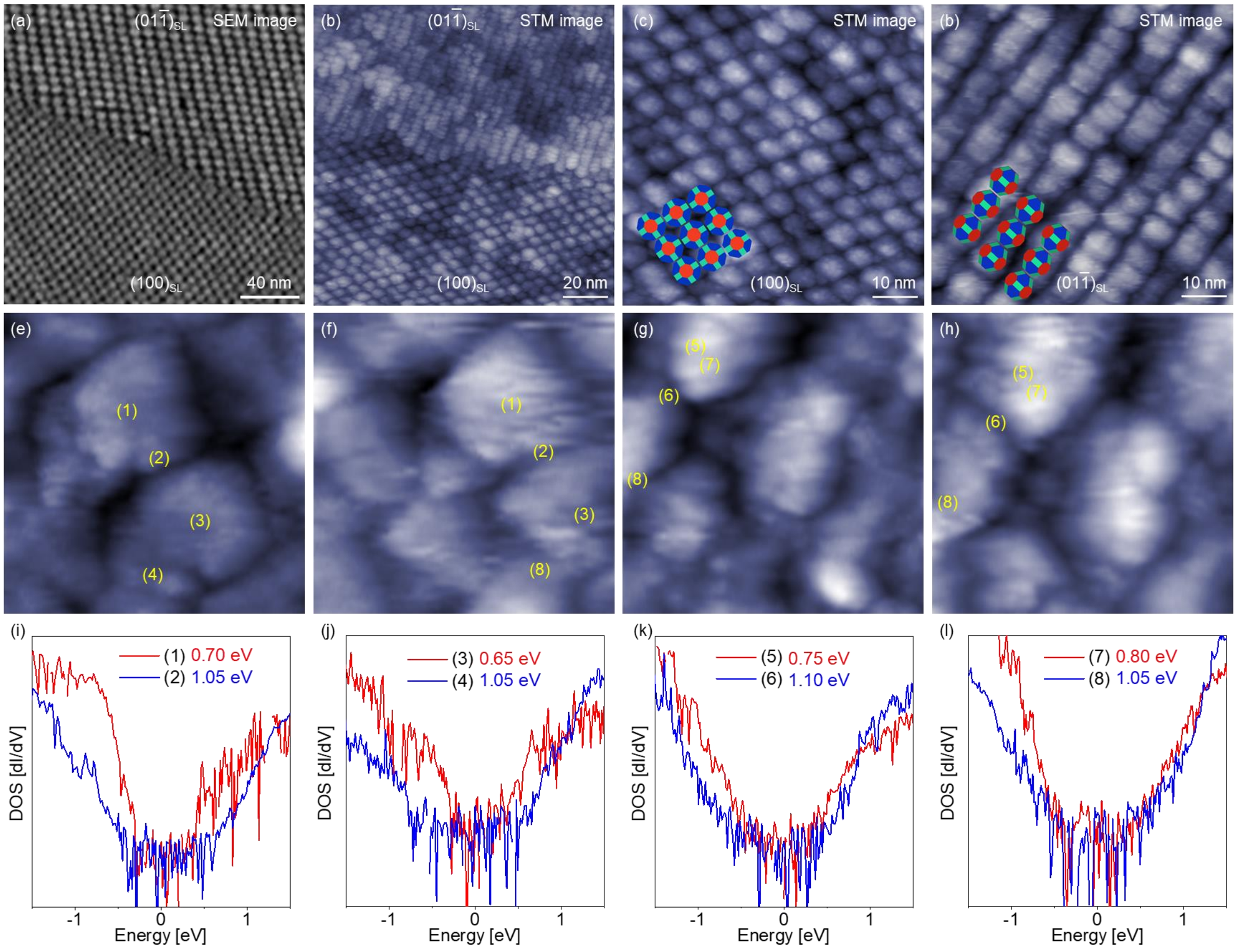

Fig. S3 Comparison of tunneling spectrums in various locations. Large area (a) SEM image and (b) STM image of PbSe QD epi-SL showing grain boundary of two orientations. It should be noted that these are two different samples. (c-d) STM enlarged images of these surfaces along with the model overlay. (e-f) STM images (20x20nm) taken in fifteen minutes apart on (100) surface shows small drift. Numbers in yellow denotes tunneling spectrums shown in semi-log plot in figure (i-j). In comparison to center of the QD, the energy bandgap is larger at the neck (the mean bandgap QD: 0.72 ± 0.05 and neck: 1.07eV ± 0.02) . STM images (20x20nm) taken in ten minutes apart on (01$\bar{1}$) surface shows small drift. Numbers in yellow show tunneling spectrums shown in figure (k-l). In comparison to center of the QD, the energy bandgap is larger at the neck. It should be noted that the unwanted surface oxidations can dope the epi-SL and result shift in fermi level as it is seen by offset in some spectra shown in (i-l). This behavior is well presented with the controlled surface oxidation using Al_2O_3 in Fig. S2.

Supplementary Discussion: Band-Edge Modeling

To fit and model the band-edge electronic structure we use the following bandgap formula, proposed by Zamani *et al*:[1]

$$I(E) = \frac{\Theta(\mathrm{E}-\mathrm{E}_g)\cdot\sqrt{\mathrm{E}-\mathrm{E}_g}}{1+e^{k(\mathrm{E}-\mathrm{E}_g)}}$$

where E_g is the bandgap energy or onset, $\Theta(E-E_g)$ is a Heaviside step function, and k is a shape parameter that controls a sigmoid function designed to reduce the intensity away from the onset of the EELS intensity. This has been shown to be effective for fitting the band-edge behavior of other narrow-gap materials with monochromated EELS.[1,2] Before fitting, we have to remove the spurious EELS signal from elastic scattering (the ZLP tail) and the continuum of states induced by carbon contamination on the sample. To remove the elastic scattering EELS, we fit a power law away from the observed onset of intensity, between 0.3-0.5 eV, and subtract it from the acquired spectrum. To remove the contamination EELS signal, we acquire a spectrum on the substrate (away from the PbSe). The contamination here should be highly comparable to the contamination on the QDs, so we treat the substrate spectrum (with the ZLP subtracted) as a rough approximation to the continuum of states induced by contamination in general and subtract it from our isolated QD and epi-SL spectra.

To fit the signal observed in the isolated QD and the epi-SL, we overlay multiple bandgap functions (of the above formula). Multiple functions are needed (even for the isolated QD) because EELS intensity can be changed both by band-edge onsets and by interband transitions, which can induce multiple features (peaks, shoulders, etc.). The justification for the number of band-edge functions used in the fitting shown in the main text is directly provided in Figure S4 and S5.

Figure S4 shows the band-edge fitting for the epi-SL spectrum with one (S4a,b), two (S4c,d), and three (S4c,d) band-edge functions. The spectral range for the ZLP fit is highlighted in blue. We can see that that the onset of EELS intensity is observed slightly past the fit region, at around 0.55 eV. We fit a bandgap function to the 0.5 eV to 2.0 eV region in Fig. S4a, and observe that the correct onset is not identified, because there is no single bandgap function that can account for both the correct onset and the higher-energy EELS intensity, demonstrating the need for multiple functions to accurately fit the spectrum. The error of the fitting (i.e., the difference between the signal and the model) is shown in Fig. S4b, along with the mean-squared error (MSE). A nominal estimate of the MSE of the measurement noise can be obtained by calculating the MSE only from the power law fit region (0.3-0.5 eV) and comparing that to the bandgap fit region (0.5-1.5 eV). The noise MSE is a factor of 5 below the fit MSE, indicating that our error is dominated by inaccuracy in the fitting, not the noise.

We can improve the MSE by fitting two bandgap functions to the spectrum. In Fig. S4c, we fit one bandgap function to the 0.5-0.75 eV region (with the goal of capturing the onset accurately) and fitting a second to the remaining spectrum (0.75-1.5 eV). The error is shown in Fig. S4d and demonstrates a significant quantitative improvement in the 0.5-0.75 eV region where the MSE is now comparable to the noise level. However, there is still quantitatively significant inaccuracy in the higher-energy portion of the fit. By adding a third bandgap function, this error can be nearly eliminated, to the extent that the MSE is comparable or superior to the noise level across the entire spectrum. It is this process that validates three-onset model shown in Figure 3.

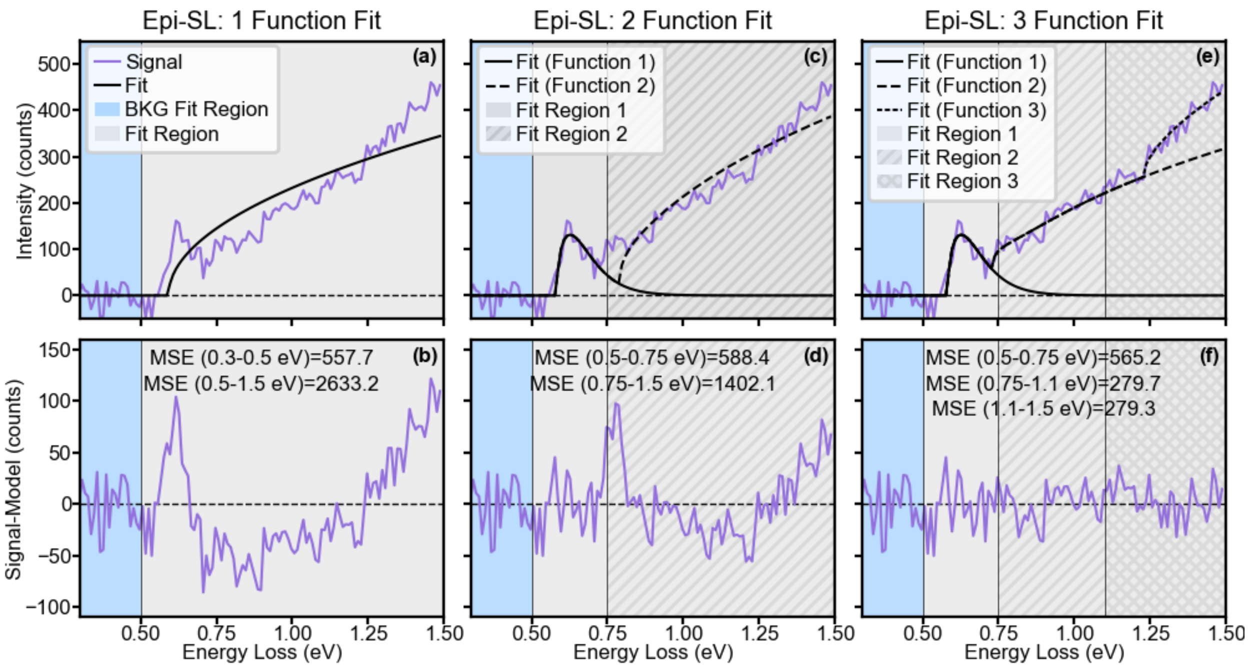

Fig. S4 Comparison of fits of the epi-SL EELS signal. (a) Epi-SL signal from Fig. 3 with a single bandgap function fit. Blue region denotes the spectral regime used to fit a power law to subtract the elastic scattering signal (0.3-0.5 eV). Gray region denotes the spectral regime used to fit the bandgap function (0.5-1.5 eV). (b) The fit error, showing a poor match both at the onset and the higher energies of the fitted regime. The MSE is calculated for the power law and the bandgap function fit regions to show the quantitative difference between error dominated by noise, and error dominated by fit inaccuracy. (c,d) The data/fit and difference of the epi-SL signal with two bandgap functions. The first function is fit across the 0.5-0.75 eV region and shows significantly improved MSE. The second model is fit to the remainder of the spectrum (0.75-1.5 eV) and still shows qualitative and quantitative inaccuracy. (e,f) The data/fit and difference of the epi-SL signal with three bandgap functions (shown in the main text). The fit qualitatively and quantitatively reproduces the band-edge response across the entire fitted region.

We perform the same analysis for the isolated QD (Fig. S5). Here, it is important to note that it is dominated by noise even in the one-function fit, as can be observed from comparing the MSE in the region of the power law fit to the MSE in the region of the band-edge fit in Fig. S5b. As a result, our fitting here is not as reliable as it was for the epi-SL spectrum, solely based on the signal-to-noise ratio. Nevertheless, in the one-function fit, we compare the MSE in the 0.5-0.75 eV range, (i.e., the spectral region used to fit the 0.58 meV onset in the epi-SL spectrum) and observe that the MSE here is much higher here than at higher energies. A two-function fit (with the same initial parameters used for the two-function fit on the epi-SL data in Figure S4c,d) is shown in Figures S5c,d. The fit results in a noticeably improved MSE in the 0.5-0.75 eV region, and more importantly, we return a strong match with the first-two functions of the epi-SL fitting. Table S1 shows the onset and shape parameters of the three-function fit for the epi-SL signal and the two-function fit for the isolated QD signal, and we see excellent agreement between both onset and shape parameter for the first two functions, confirming that the band-edge fine structure is similar in the isolated QD and epi-SL below the neck bandgap. We note that here the improvements are below the level of the noise MSE (unlike the more definitive fitting in Fig. S4), but the

combination of the improved MSE in the 0.5-0.75 eV region and the match to the epi-SL fit parameters motivate us to use this fitting in the main text.

Band Edge Function	Onset (E_g)	Shape Parameter (k)
Isolated QD Onset 1	0.53 eV	16.9
Isolated QD Onset 2	0.76 eV	~0 (<< 0.1)
Epi-SL Onset 1	0.58 eV	17.2
Epi-SL Onset 2	0.73 eV	~0 (<< 0.1)
Epi- SL Onset 3	1.23 eV	0.2

Table S1. Function Parameters for Band-Edge Fitting shown in Fig. 3

We also consider the possibility that the 1.2 eV feature observed in the epi-SL spectrum is not the band-edge onset of the QD necks but instead an interband transition effect that is masked by noise in the isolated QD spectrum. To prove otherwise, we perform a three-function fit of the isolated QD spectrum (shown in Fig. S5e,f). Here, the addition of a third band-edge function does result in a much smaller change in MSE than the addition of the second band-edge function. Moreover, the parameters of the third function (E_g = 1.38 eV, k = 3.3) in the isolated QD spectrum do not show the same level of agreement to the third fitting model of the epi-SL data that we observed in the first two fitting models in Table S1. As a result, the addition of the third fitting model does not appear to be statistically significant, and in combination with other validation shown in the supplementary information and the main text, confirms that the 1.2 eV onset represents a new feature of the electronic structure of PbSe QDs induced by the epitaxial necks that is not observed in isolated QDs.

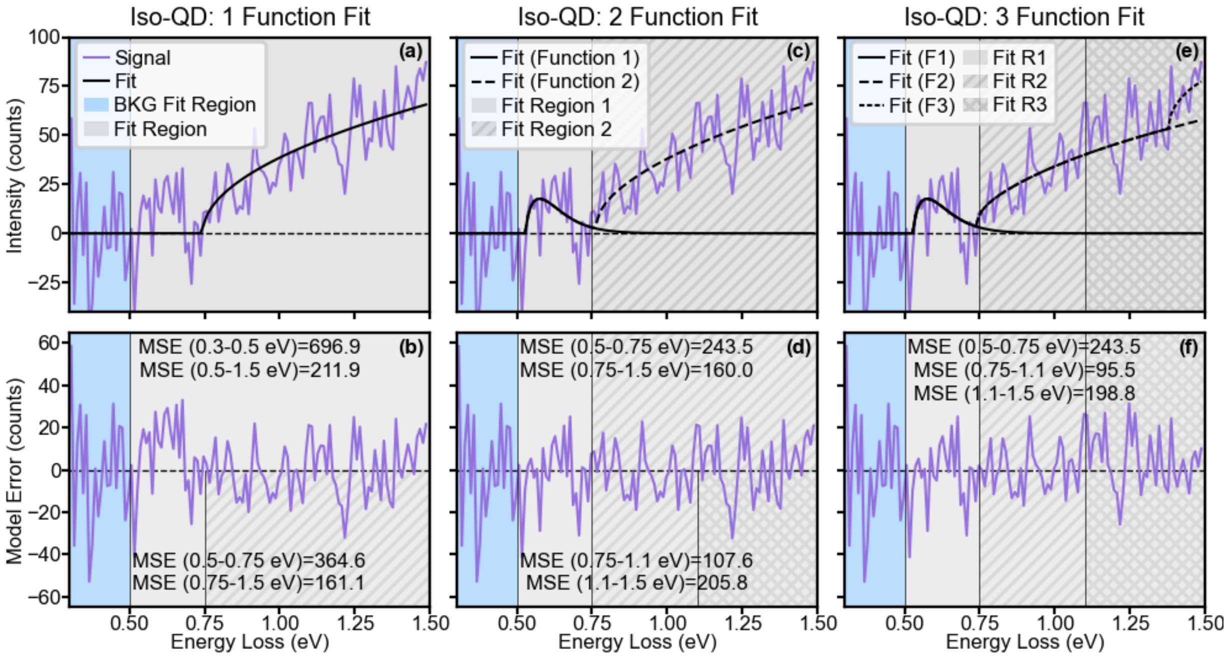

Fig. S5 Comparison of fits of the isolated QD EELS signal. (a-f) The identical analysis shown in Fig. S5 but for the isolated QD signal from Fig. 3 in the main text. Here, the MSE is dominated by the noise (as seen by comparing the MSE in the blue region used to fit the power law to the MSE in the gray regions used to fit the functions). However, by comparing the MSE in the same

spectral regions in a one-function fit (a, b) to a two-function fit (c, d) we can demonstrate that including a second model fit in the 0.5-0.75 eV region returns an improved MSE (along with fit parameters in agreement with the epi-SL modeling of the QD core onset, see Table S1). A three-function fit is also performed (e, f), but this only slightly improves the fit quantitatively and does not appear to improve the fit qualitatively, so we believe that this model is an overfitting of noisy data, not an accurate representation of the electronic structure.

It should be noted that this strategy involves multiple levels of approximation and several significant hurdles that make precise measurements a challenge. First, it is well established that the ZLP tail (especially for monochromated EELS) does not follow power law decays over large ranges[3], especially where the ZLP still has strong intensity. For the band-edge modeling we only consider a small spectral range (0.3-1.5 eV), so the power law should be a good (but not perfect) approximation to the elastic scattering signal. Second, we perform spectrum-by-spectrum normalization on the SIs (as described later in Figure S7), that should largely account for any changes to the EEL intensity that are simply due to thickness related effects (either the presence of QDs or variations in the contamination on the substrate). However, such effects not only affect the EEL signal quantitatively, but can change EEL signal qualitatively as well (i.e., effectively broadening the ZLP). We believe the validation shown in Fig. S5f and S5g, shows that these qualitative differences are minimal, but this is another potential channel for inaccuracy in the onset measurements with EELS.

The other major problem is the actual band-edge fine-structure of PbSe itself. For instance, the onset of the SiO_2 at 8.5 eV exhibits a massive sharp wall that allows for robust fitting, in a way that the above approximations would not be likely to influence. How sharp the onset of EELS intensity is at the band edge is a function of the joint-density of states at the bandgap and varies from material to material, and it is unfortunately quite weak for PbSe. As a result, we lack a robust sharp wall, like is obtained from SiO_2, and are hence much more sensitive to the subtle inaccuracies introduced by our fitting methodology. As a result, the fitting parameters are sensitive to the fit regions and the initial parameters of the fit. Presented here are the best qualitative and quantitative fits achieved by wide parameter sweeps, and we observed that different fitting inputs do change the actual values of the onsets and the shape parameters of the fitted band-edge functions. However, there are no fits in our parameter sweep, with minimized MSE, where the fitted values do not corroborate the observations stated in the above analysis and the main text of the manuscript: a low-energy band-edge (onset between 0.5-0.8 eV) that is observable in both the iso-QD and the epi-SL and a high-energy band-edge (onset >1.1 eV) that is only observable in the epi-SL. Thus, while we do not believe that the EELS fitting here constitutes an accurate measurement of the exact bandgap value, potentially explaining why the EELS and STS bandgap values differ slightly, we do believe it serves as a confirmation of the two band-gaps observed in STS.

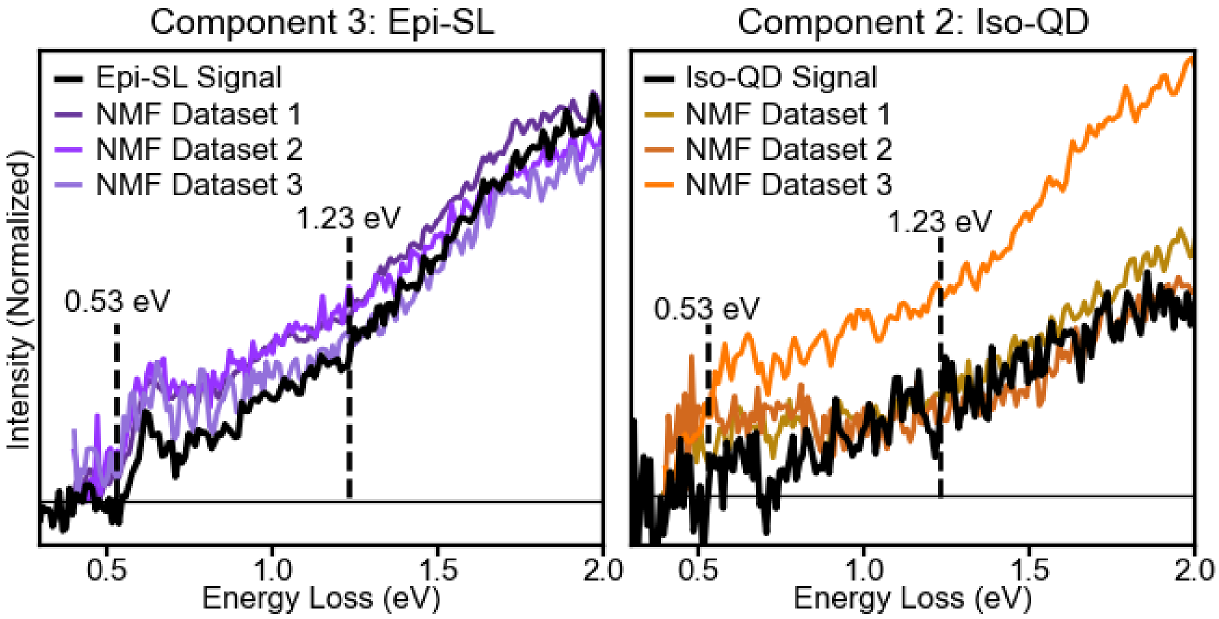

Fig. S6 Comparison of band-edge structure in NMF components and raw spectral analysis. The general shape of the NMF components is shown in Figure 4 and compared to the epi-SL and iso-QD signals from Fig. 3. The Component 2 spectral end members most strongly resemble the isolated QD signal, while the Component 3 spectral endmembers resemble the epi-SL signal. In the Component 3 spectra, we can clearly see all three NMF components exhibit the same 0.53 eV onset, shown in Fig. 3. Furthermore, the high-energy behavior (with the secondary onset at ~1.2 eV) is also well matched. In the Component 2 spectra, we see that NMF 1 and NMF 2 nicely match the band-edge structure of the iso-QD signal. They exhibit an onset of intensity at ~0.53 eV (which is more difficult to observe due to the worse signal-to-noise ratio obtained in the iso-QD measurement) without the QD neck onset at 1.2 eV. The exception here is the NMF 3 component, which shows electronic structure similar to the epi-SL signal and the Component 3 NMF spectra. This occurs because the Dataset 3 in Figure 4 does not possess a significant fraction of iso-QDs. As a mathematical deconvolution technique, NMF can split and/or mix different physical mechanisms in a single component. Since there is an iso-QD to epi-SL ratio of ~1:1000 in Dataset 3, (compared to 1:16 in Dataset 1, and 3:8 in Dataset 2) it is not surprising that the epi-SL electronic structure mixes into the NMF component describing the iso-QD behavior.

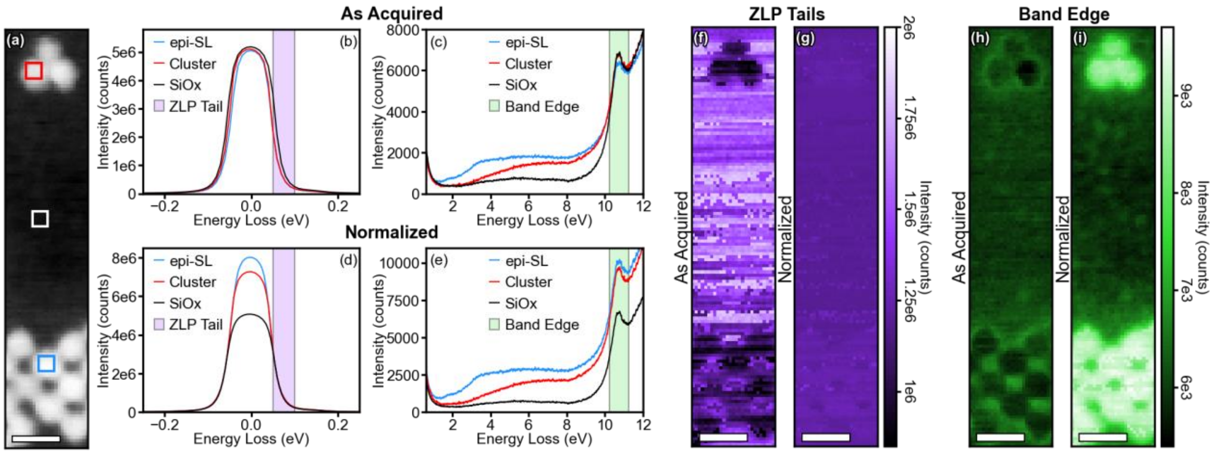

Fig. S7 Normalization of the EEL spectra. For three-dimensional samples, where thickness changes significantly, it is important to normalize the EEL spectra due to changes in the inelastic scattering cross section (especially here for heavy materials such as lead). However, we acquire with long dwell times at high beam currents resulting in a saturated spectrum, which makes robust normalization difficult. The problem is exemplified in (a-c) The HAADF image of the trimer cluster near an epi-SL shown in (a), where we acquire representative spectra from 3 different regions: the trimer cluster (red), the epi-SL (blue), and the substrate (white). These representative spectra are plotted to show the zero-loss peak (ZLP) in (b) and the electronic structure in (c). The problem with saturation can be seen in the ZLP, all three spectra have the same overall intensity despite all being acquired in the same dataset. The reason for this is that all three spectra are acquired in the same probe condition with the same nominal full width at half maximum (FWHM) of the ZLP but are saturated to different degrees. Since the epi-SL and trimer spectra pass through the thick PbSe the total EELS cross section is increased, meaning the ZLP here is saturated less and we see a spectrum closer to the true FWHM of the probe. While this difference is minimal, it results in unphysical comparisons in the electronic structure as seen in (c). Here, if we look specifically at the peak at 11 eV corresponding to the SiOx band edge from the supporting membrane, we see that even though the probe passes through additional material and should have additional transitions available to probe when interacting with the substrate and a QD the intensity is identical or less than observed in just the substrate. To remedy this problem, we normalize to the tails of the ZLP outside of the saturated region. Since the probe condition is nominally the same throughout the entire SI, we believe that the tails of the ZLP should be nominally identical in each pixel of the SI. Thus, we follow the procedure outlined in literature to align the ZLP tails of every single pixel in the spectrum[3]. The results for the ZLP and electronic structure after normalization are shown in (d) and (e) respectively. Here we see that now the epi-SL and trimer cluster ZLP intensities are much higher than the substrate ZLP, since these spectra were reduced in intensity due to passing through the heavy PbSe. Furthermore, now we see that the epi-SL and trimer spectra are everywhere greater than the substrate spectrum, which is important because the additional material should result in additional energy loss and the substrate spectrum should be considered a background that could effectively be subtracted off to examine only the contribution from the PbSe QDs. We avoid this in the main text because the substrate spectrum is almost entirely flat in the region of interest, so nothing is gained from looking at the background subtracted spectrum compared to the normal spectra. The benefit of the normalization is visualized

in (f-i) by looking at the average intensity in the ZLP tail region we use to normalize and at the band-edge onset. In the as-acquired ZLP tail (f) we see that the intensity is much higher outside of the QDs than inside them, due to the changes in the inelastic scattering cross section, while in the normalized ZLP tail (g) the plot is smooth consistent with a single probe-condition throughout the whole experiment. Then in the as-acquired band-edge image (h) we see the plot is only bright around the edges of the PbSe QDs, meaning as it interacts with the edge it starts to pick up the additional transitions due to the electronic structure of the PbSe, while the PbSe is still extremely thin, but then as the probe hits the full thickness of the PbSe the intensity is reduced. In the normalized band-edge image (i) we see that the new electronic structure of the PbSe increases the total observed signal throughout the cluster and epi-SL.

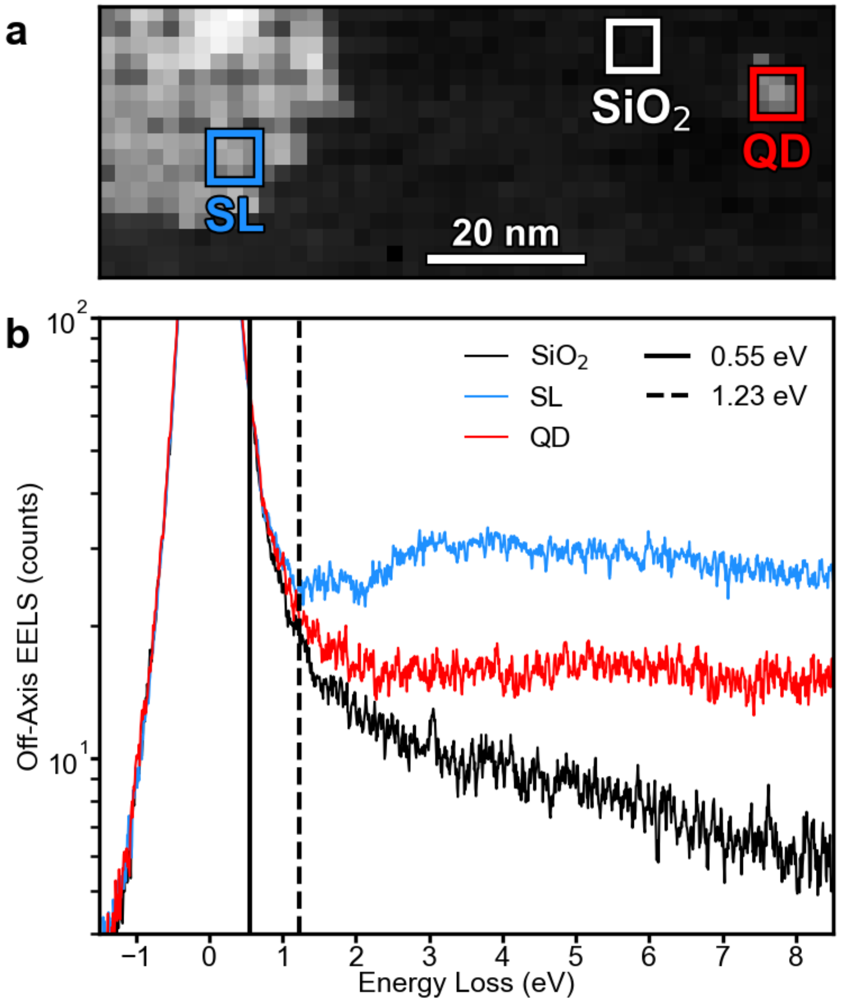

Fig. S8 Off-Axis EELS Analysis. As stated in the main text the new electronic states induced by the epitaxial connectivity can be excited in the aloof mode, allowing significant intensity to be observed in the vacuum. By using an off-axis EELS collection configuration it is possible to cut-out the aloof signal to achieve only a highly localized signal[4]. (a) In the off-axis EELS geometry we find a region akin to the region analyzed with on-axis EELS in Fig. 3 with an iso-QD in close proximity to an epi-SL. The HAADF reference is shown in (a) with the representative spectra in (b). Here we clearly, see a new onset of intensity at ~1.23 eV that is present in the epi-SL but not the iso-QD, in the absence of 'aloof' excitation. The 0.55 eV onset is harder to see due to the extremely low signal involved with off-axis EELS, but we can see that just before the QD neck onset (i.e., the spectral range between 0.7 eV and 1.1 eV) the QD spectrum is clearly higher in intensity than the background SiO_2 spectrum, indicating that there is additional electronic structure detected (corresponding to the lower band-gap onset of the QDs). Due to the noisiness of the off-axis measurements we use the on-axis measurements in the main text, but these results demonstrate that the conclusions in main text are not influenced by delocalization.

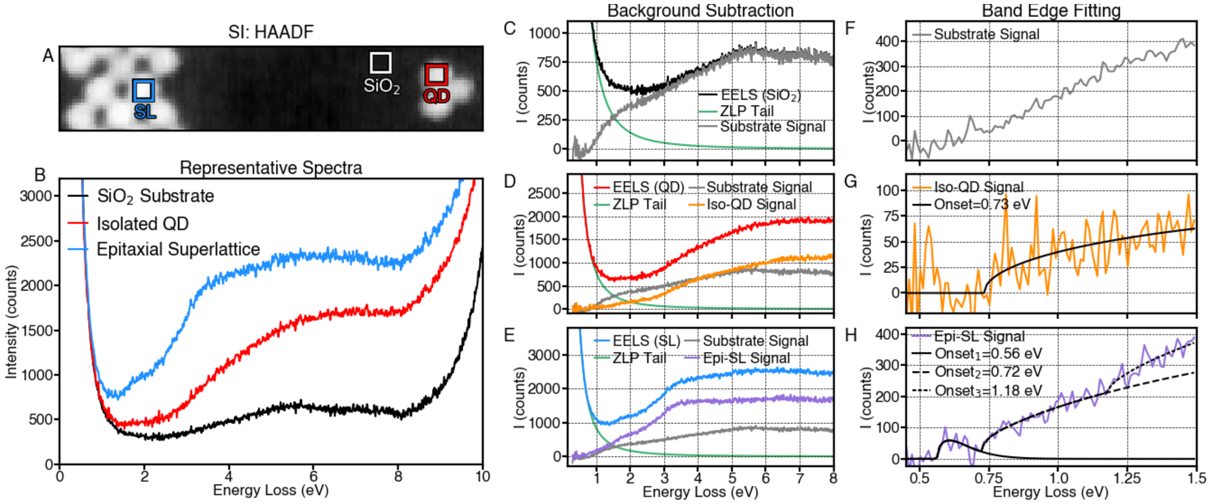

Fig. S9 Band Edge Modeling for Dataset 2. A comparable analysis for the band edge modeling to that shown in Figure 3 of the main text for the trimer cluster dataset highlighted in Figure 4 of the main text, showing the same electronic structure for both datasets. (**a**) HAADF reference image from the SI. (**b**) Three representative spectra from different regions. (**c-e**) The representative spectra from (**b**) with a power law background subtraction. The substrate signal in (**f**) as a reference. The fitted band-edges of the trimer cluster in (**g**) and epi-SL in (**h**). The values here for the epi-SL, are extremely close to the values from the main text figure, further demonstrating that these onsets are a fundamental aspect of the epi-SL electronic structure. For the iso-QD signal, the second 0.73 eV onset matches extremely well, in both onset energy and shape, to the 0.72 eV onset in Figure 3, indicating a consistent electronic structure between the iso-QD observed there and the trimer cluster here which possesses no epitaxial necking. However, the first onset at 0.51 eV, while matching well with the 0.53 onset energy in Figure 3, has an extremely narrow shape factor, indicating that this is likely fitting a spike in the noise in the acquisition as opposed to the genuine onset of intensity. It is important to emphasize that for both this dataset and the one modeled in Figure 3, that the low SNR in the spectra and the approximations needed to account for the continuum of states from the substrate make it very difficult to obtain an accurate measurement of the band-edge onset. In the epi-SL where the signal is higher, the onset at 0.56 eV is much more deterministic, but in the isolated QDs the signal is too low to obtain an unambiguous measurement. The key takeaway should not be the value of the first onset, but rather the similarity in shape of the spectra between the iso-QDs in Figure 3 and here, and the absence of the last high energy onset above near the band-edge of the QD necks. The similarity between the band-edge structures shown here demonstrates the repeatability of the measurement.

References

1 Zamani, R. R. *et al.* Unraveling electronic band structure of narrow-bandgap p–n nanojunctions in heterostructured nanowires. *Physical Chemistry Chemical Physics* **23**, 25019-25023, doi:10.1039/D1CP03275E (2021).

2 Dimitrievska, M. *et al.* The Advantage of Nanowire Configuration in Band Structure Determination. *Advanced Functional Materials* **31**, 2105426, doi:https://doi.org/10.1002/adfm.202105426 (2021).

3 Hachtel, J. A., Lupini, A. R. & Idrobo, J. C. Exploring the capabilities of monochromated electron energy loss spectroscopy in the infrared regime. *Scientific Reports* **8**, 5637, doi:10.1038/s41598-018-23805-5 (2018).

4 Hoglund, E. *et al.*
